# Humoral antimalaria immune response in Nigerian children exposed to helminth and malaria parasites

**DOI:** 10.3389/fimmu.2022.979727

**Published:** 2022-09-02

**Authors:** Selorme Adukpo, Ayodele Adedoja, Meral Esen, Michael Theisen, Francine Ntoumi, Olusola Ojurongbe

**Affiliations:** ^1^ Department of Pharmaceutics and Microbiology, School of Pharmacy, University of Ghana, Accra, Ghana; ^2^ Institute of Tropical Medicine, University of Tübingen, Tübingen, Germany; ^3^ Department of Medical Microbiology and Parasitology, Ladoke Akintola University of Technology, Osogbo, Nigeria; ^4^ Department of Medical Microbiology and Parasitology, University of Ilorin Teaching Hospital, Ilorin, Nigeria; ^5^ Department for Congenital Disorders, Statens Serum Institut, Copenhagen, Denmark; ^6^ Centre for Medical Parasitology at the Department of International Health, Immunology, and Microbiology, University of Copenhagen, Copenhagen, Denmark; ^7^ Infectious Disease Department, Fondation Congolaise pour la Recherche Médicale, Brazzaville, Republic of Congo; ^8^ Centre for Emerging and Re-emerging Infectious Disease, Humboldt-Bayer Foundations Research Hub, Ladoke Akintola University of Technology, Ogbomoso, Nigeria

**Keywords:** malaria, helminths, immunology, antibodies, *hymenolepis nana*, hookworm

## Abstract

**Background:**

Malaria and helminthic parasites are endemic in tropical countries, and co-infections might influence host-parasite interactions. In this community-based cross-sectional study, the effect that the presence of soil-transmitted helminths (STH) (Hookworm, *Hymenolepis nana)* and *Schistosoma haematobium infections* could have on the immunoglobulin (Ig) candidate protein of the malaria vaccine GMZ2 levels was evaluated.

**Methods:**

Blood, stool, and urine samples were collected from 5-15-year-old children to diagnose *P. falciparum* (Pf), STH, and *Schistosoma haematobium*, respectively. Identification and quantification of the parasite load of STH and *S. haematobium* were achieved by light microscopy. A polymerase chain reaction was carried out to detect submicroscopic infections of *P. falciparum*. Plasma levels of GMZ2 specific IgG and its subclasses were quantified by ELISA.

**Results:**

The median level of total IgG in individuals with co-infection with Pf/*H*. *nana* was significantly lower in the mono-infected group with Pf (p = 0.0121) or study participants without infection (*p*=0.0217). Similarly, the median level of IgG1 was statistically lower in Pf/*H. nana* group compared to Pf-group (*p*=0.0137). Equally, the Pf/*H. nana* infected individuals posted a lower level of IgG1 compared to Pf-group (p=0.0137) and IgG4 compared to the Pf-group (*p*=0.0144). Spearman rank correlation analyses indicated positive relationships between the densities of *H. nana* (ρ=0.25, *p*=0.015) and *S. haematobium* (ρ=0.36, *p<*0.0001).

**Conclusions:**

Hookworm and *H. nana* infections are associated with reduced GMZ2 specific IgG levels. This study shows the possible manipulation of immune responses by helminths for their survival and transmission, which may have serious implications for vaccine development and deployment in helminth-endemic regions.

## Summary

Geographically, malaria and infections caused by worms, including intestinal worms and liver flukes, overlap greatly in distribution, and hence there are instances where these parasites infect the same person simultaneously. Pockets of studies indicate that these worms may have an antagonistic effect on the immune response to malaria when coinfected, while others suggest a protective effect, especially against the severe form of malaria. Therefore, this study looked at how intestinal worms such as dwarf tapeworm, hookworm, *and Schistosoma haematobium* that causes bilharzia could influence antibody levels of GMZ2, a malaria-specific antigen that is being developed into a vaccine. The results suggest that hookworm and dwarf tapeworm, a less fancied cestode, infections significantly reduce levels of GMZ2 antibodies in individuals co-infected with malaria parasites and any of these worms. This may compromise the effectiveness of antibody production and antibody-mediated immune response to malaria in individuals with these parasites or the development of GMZ2-mediated immunity against malaria in individuals vaccinated with GMZ2 while infected with any of these parasites. Therefore, the study shows a manipulation of immune responses by these worms for their survival and transmission, which can have profound consequences for the development and deployment of the malaria vaccine in endemic regions.

## Introduction

Helminth infections caused by *Schistosoma* spp and soil-transmitted helminths (STH), such as *Ascaris lumbricoides*, hookworm, and *Trichuris trichiura*, are highly prevalent in developing countries. These parasites are responsible for more than 40% of worldwide morbidity from all tropical parasitic infections other than *Plasmodium* spp (1). The geographical distribution of helminths overlaps with malaria parasites, making multiple infections among these parasites a common occurrence (2, 3). Another widely distributed helminth whose infection seems underappreciated is the dwarf tapeworm, *Hymenolepis nana*.

Several Plasmodium species cause malaria in humans: *P. falciparum*, *P. vivax*, *P. ovale*, *P. malariae*, *P. knowlesi* and *P. cynomolgi* (4–6) with *P. falciparum* (Pf) responsible for the most severe form of the disease. However, in the human host, *Pf* and helminths occupy different niches, and any interaction between them may be indirect. This interaction may occur through the influence each of them has on immune responses. Malaria elicits a non-sterile immunity in age and exposure-dependent manner, first against severe malaria and later against uncomplicated malaria after repeated infections (7). This semi-immunity holds in check the asexual growth of the parasite and is believed to be mediated by antibodies, especially cytophilic immunoglobulin (Ig) G1 and IgG3 (8–11). These antimalarial antibodies act independently to block merozoites’ invasion of erythrocytes and, in conjunction with blood leukocytes, to retard parasite growth (12–14), as evidenced in parasite growth inhibition and antibody-dependent cell inhibition assays (12, 14). Several *Pf* antigens elicit functional antibody responses and are being investigated as antimalaria vaccines. One of such molecules is GMZ2, a glutamate-rich protein fusion protein and the merozoite surface protein 3 of *Pf* merozoite (15). IgG, particularly the IgG1 and IgG3 subclasses, directed against the individual components of this protein, are associated with reduced incidence of clinical malaria in populations naturally exposed to malaria (8–10, 16).

Epidemiological evidence points to a disproportionate prevalence of *Pf* infection in children infected with STHs compared to non-infected ones (17–20) possibly because of the negative effect of the various helminth immune response to malaria parasite in those individuals. However, there is limited information on how specific STHs shape the humoral immune system of antimalaria (21–24). In this regard, the current community-based cross-sectional study in children was designed to evaluate the relationship between helminth infection and anti-GMZ2 specific IgG responses, knowing that this population is developing its immunity against malaria and is the most vulnerable to both parasite infections

## Materials and methods

### Study site, sample collection, diagnosis, and immunological assays

The study utilized samples collected in Pategi and Lafiagi communities in Kwara State, North Central Nigeria from October 2012 to May 2013, which traversed dry and rainy seasons. The Kwara State Ministry of Health Ethical Committee approved the study, with the ethical certificate reference number being MOH/KS/777/41. Whole blood, stool and urine samples were collected from participants (children between 5 and 15 years) whose parents/guardians gave verbal informed consent after the study procedures, samples to be taken, study benefits, potential risks, and discomforts were explained. In addition, assent was obtained directly from the participating school children. The study participants included only children between the ages of 5 and 15 years who were asymptomatic for malaria and helminthiasis and showed no signs of illness. The parasite burden for STH that included *H. nana* and hookworm (*Ancylostoma duodenale* and *Necator americanus*) was calculated as the number of parasites per gram of stool, the number of parasite eggs per 10 ml of urine in the case of *S. haematobium*, and the density of *P. falciparum* was evaluated as the parasite per microlitre of blood. However, the details of the study site, sample collection, and diagnosis are described elsewhere (17). Total IgG and its subclasses (IgG1, IgG2, IgG3, and IgG4) against GMZ2 were quantified in plasma from children using the Afro Immuno Assay (AIA) ELISA protocol with slight modifications (9, 10). Briefly, the GMZ2 antigen (Kindly provided by Michael Theisen), diluted in 1X phosphate-buffered saline (PBS) was adsorbed on the bottom and inner surfaces of the wells of 96-well microtiter ELISA plates (Maxisorp Nunc, Denmark) at 500 ng/mL and 100 μL/well. Plates were incubated overnight at 2-8°C, followed by a blocking buffer prepared as 5% Marvel skimmed milk powder (CREED Family Butchers, UK), 0.1% Tween-20 (Bio SB, Santa Barbara, USA) in PBS (Gibco Inc, USA) at 150 μL incubation at room temperature for one hour. Plasma samples diluted 1: 200 (for total IgG) and 1:50 (for IgG subclasses) in serum dilution buffer (PBS with 1.0% Marvel skimmed milk powder and 0.1% Tween-20) were added in duplicates at 100 μL/well, followed by another round of incubation at room temperature for two hours. Serially diluted antibodies, negative control plasma obtained from five European blood donors who had never been exposed to malaria according to their travel records, and a buffer blank (1.0% Marvel Skimmed milk powder and 0.1% Tween-20 in PBS) were included on each plate to generate a standard curve for transforming optical densities (ODs) into a concentration unit. A 1:50,000 dilution of goat anti-human IgG (H+L) conjugated to horseradish peroxidase (HRP) (A1881, Invitrogen, Carlsbad, CA, USA) was added to each well for total IgG detection. IgG subclasses were detected using HRP conjugated sheep anti-human IgG1 (AP006) (1:10000), IgG2 (AP007) (1:2000), IgG3 (AP008) (1:15,000) and IgG4 (AP009) (1:2000) antibodies (The Binding Site Group Ltd, Birmingham, UK), respectively. All the secondary antibodies were diluted in the serum dilution buffer before adding to appropriate plates at 100 μL/well and incubated for one hour at room temperature. The plates were washed four times between consecutive steps using a washing buffer (0.1% Tween 20 and 0.5 M NaCl in PBS). One hundred microliters of 3,3′,5,5′-tetramethylbenzidine substrate (Kem-En-Tec Diagnosis A/S, Taastrup, Denmark) were dispensed into each well and incubated for 30 minutes in the dark. Color development was stopped by adding 100 uL of 0.2N H_2_SO_4_ and the ODs read immediately at 450/620 nm using BioTek 800 TS Absorbance Reader (Winooski, USA). The OD values for the test samples were converted into antibody concentration with the standard reference curves generated for each plate using the predetermined concentrations of the serially diluted standards and their corresponding OD values. A four-parameter curve fitting software (ADAMSEL, version 1.1 build 40^©^ 2009 EJ Remarque) was employed in the generation of the standard curve and transformation of the ODs of the samples into concentration units.

### Data analysis

Statistical analyses were performed using the GraphPad Statistical software package version 8.0 (GraphPad Software, Inc., CA, USA). Differences between three or more groups were evaluated by Kruskal-Wallis one way analysis of variance followed by Dunn correction for multiple comparisons as the data was not normally distributed. Spearman rank correlation analysis was used that ranks data points and determines the strength and direction of the relationship between two ranked variables to assess the relationship between parasite load/burden and immunoglobulin levels as the data were not normally distributed. For all tests, the difference between the groups was considered statistically significant at p<0.05.

## Results

### Study participants

A total of 450 children were enrolled in this study. The children were 5-15 years with a mean age of 9.5 years. Of these, 220 (48.89%) were females, and 230 (51.11%) were males. For statistical analyses, study participants were grouped as those who were not infected with any parasites at the time of sampling (No infection), those who were infected with only *Pf*, those with *H. nana* mono-infection (Hn), those with Hookworm (*Necator americanus* and *Ancylostoma duodenale*) single infection (Hw), single *Schistosoma haematobium* infection (Sh), those with coinfections of *Pf* and *Hn.*, *Pf* and Sh, *Pf* and Hw coinfections. However, groups that included multiple infections with only helminths were excluded from the analyses. Of the 450 children, 44 (9.78%), 47 (10.44%), 24 (5.33%), and 157 (34.89%) tested positive for *Pf*, Hw, *Hn*, and *Sh*, respectively. Study participants with Pf/Hw, Pf/Hn, and Pf/Sh are, respectively, 11 (2.44%), 14 (3.11%), and 52 (11.56%), while those with three or more infections are 33 (7.33%). Only 68 (15.11%) children tested negative for all parasites.

### Distribution of the various parasites

**Figure 1:**
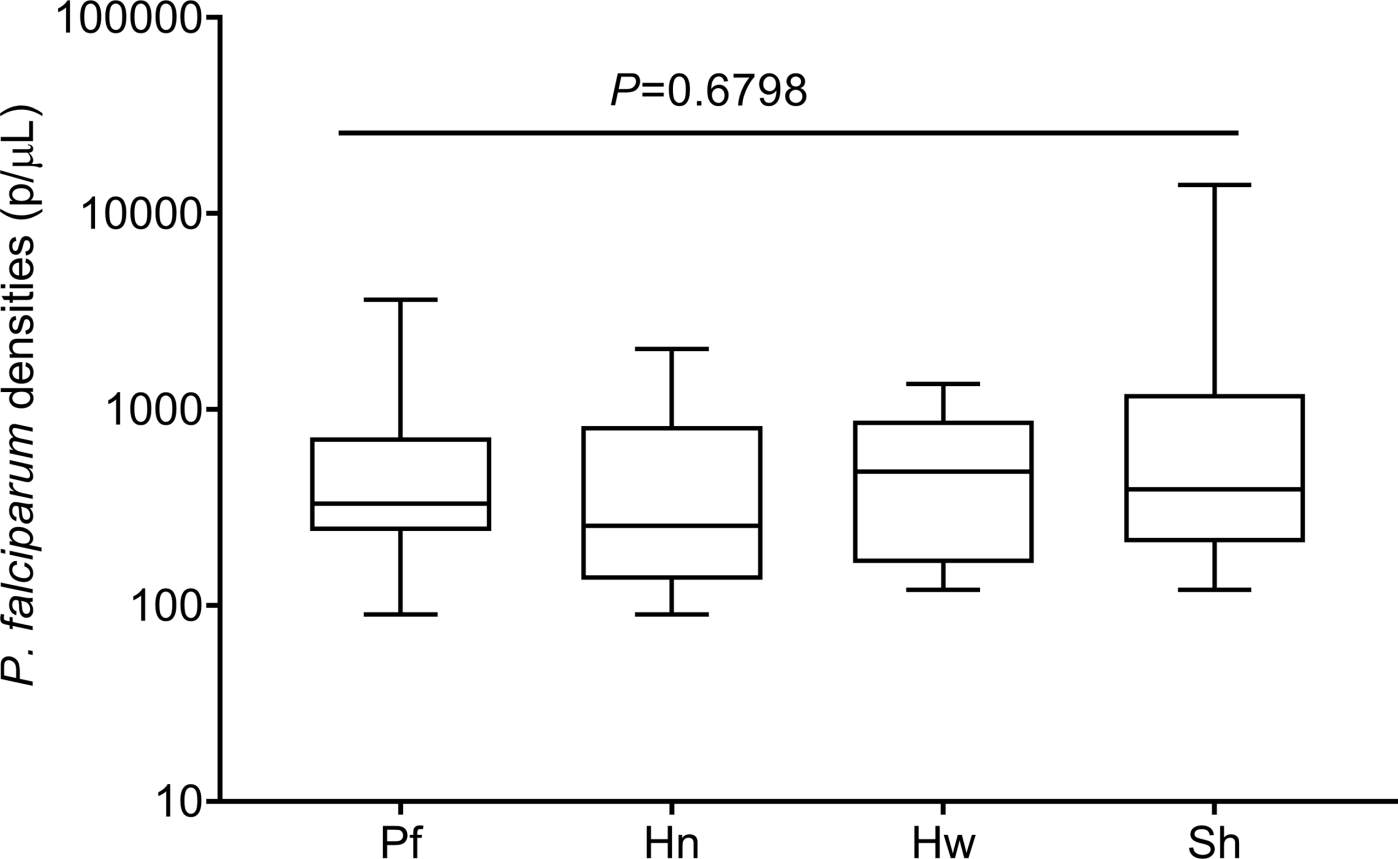
Levels of *Plasmodium falciparum* parasite densities in mono and concomitant infections with helminthic parasites. The data are presented as a box plot with the lower and upper whiskers denoting the lowest and highest *P. falciparum* densities in each group, respectively. The p-values were generated from Kruskal-Wallis analysis of variance on rank with significant level set at *p* < 0.05.

*P. falciparum* densities during mono-infection and the presence of various helminths were statistically similar (**Figure 1**) as analyzed by Kruskal–Wallis analysis of variance (ANOVA) as the data was not normally distributed. Likewise, the level of each helminth during mono-infection did not differ from the level when co-infected with malaria (**Table 1**). Parasite data has been previously, presented and discussed (17).

**Table 1:** Burden of helminths during mono- and co-infections with Plasmodium falciparum.

Infection type	*H. nana*	Hookworm	*S. haematobium*
Mono Infection	400 (200.0-110.0)	1200 (1000-1400)	77 (49.5-97.0)
Co-infected with malaria	500 (200-1200)	1200 (1200-1400)	80 (58-97.5)
*P-value*	0.295	0.3827	0.1337

The median values of the density/load/of the egg or larvae of the various helminthic parasites are presented with the interquartile range in parentheses.

### Plasma levels of total IgG to GMZ2 in study participants

**Figure 2:**
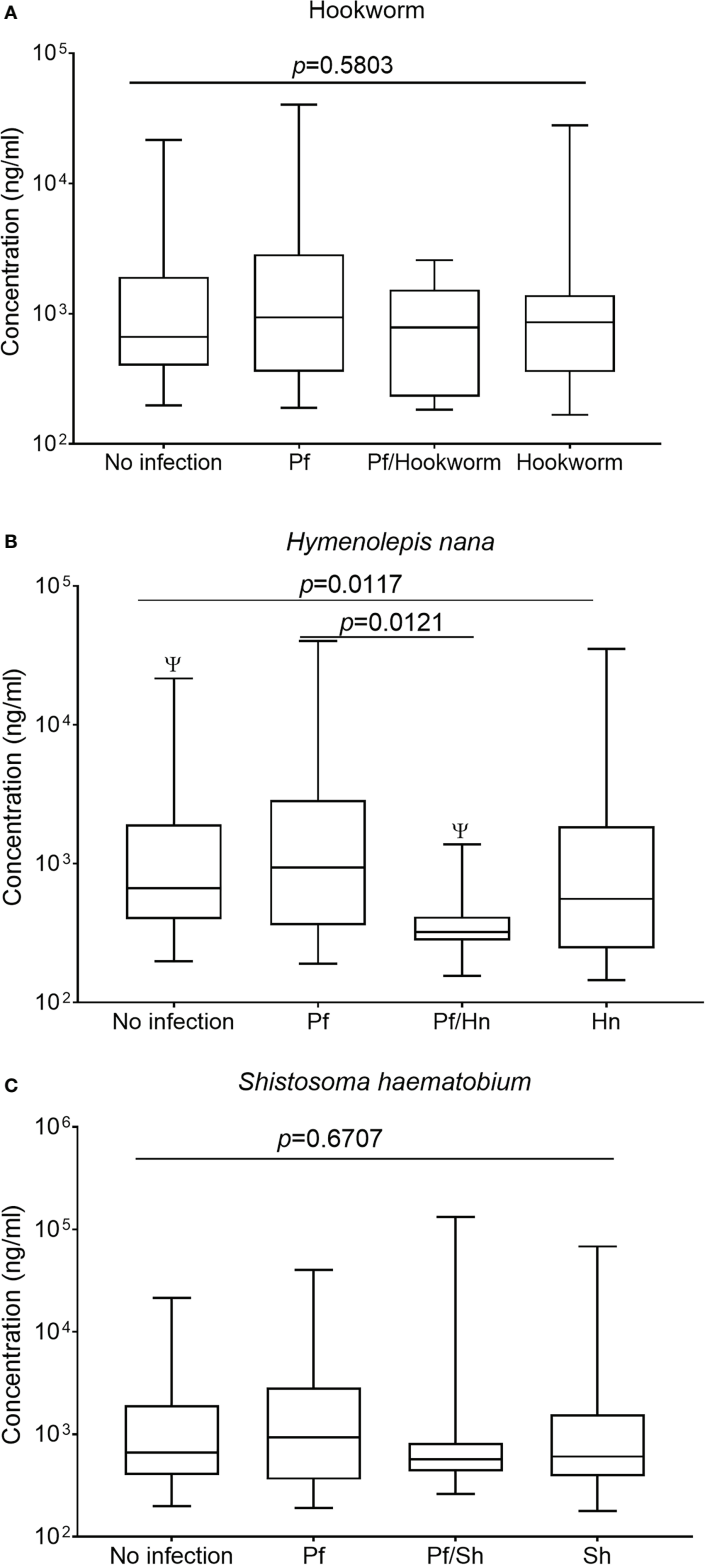
*Hymenolepis nana* infection is associated with reduced levels of IgG in GMZ2. Plasma levels of IgG to GMZ2 in children without parasitic infection (No infection), with *P. falciparum* (Pf), hookworm, *H. nana*, or *Schistosoma haematobium* (Sh) infection presented as a box plot. The line through each box represents the median antibody value; the edges of the box indicate the interquartile range, with the lower whisker showing the minimum while the upper one denotes the maximum antibody level in each group. Graph **(A)** is for Hookworm infection, graph **(B)** is for *H. nana*, and graph **(C)** is for *S. haematobium*. Kruskal-Wallis analysis of variance on rank followed by multiple comparison tests revealed significant differences between No Infection and Pf/Hn (*p* = 0.0217).

The supplementary figure shows the median levels of total anti-GMZ2 IgG among the study groups after Kruskal–Wallis ANOVA evaluation. No effect of helminthiasis on antibody levels was detected when all anti-GMZ2 specific IgG from Hookworm, *H. nana*, and *S. haematobium* were grouped together as helminth infection and those of the helminth-malaria co-morbidity were also grouped separately and median levels of total IgG, IgG1-4 were compared separately among groups (**Supplementary 1**). Regrouping the data and analyzing each helminth separately revealed more information on the effect of the helminths on the antibody levels hence further analyses or comparisons were carried out according to the No Infection, Pf, Hw, Sh, Pf/Hn, Pf/Hw and Pf/Sh groups. The median level of total IgG was significantly lower in participants with Pf/Hn co-infection (320.5 ng/ml; IQR, 279.9-414.6) compared to those infected with Pf alone (935.6 ng/ml, IQR 359.4-2866; *p*=0.0121) or study participants who were parasite-free at the time of sampling (662.8 ng/ml, IQR, 398.4-1915; *p=*0.0217) (**Figure 2B**). IgG levels in both the Pf/Hw co-infections (783.2 ng/ml, IQR, 230.8-1535.0) and the Pf/Sh co-infections (570.7 ng/ml, IQR, 434.5-821.0) were generally lower than those of Pf (935.6 ng/ml, IQR 359.4-2866) or the No infection group (662.8 ng/ml; 398.4-1915.0) but the difference was not significant (**Figures 2A, C**).

### Anti-GMZ2 IgG subclasses in study participants

**Figure 3:**
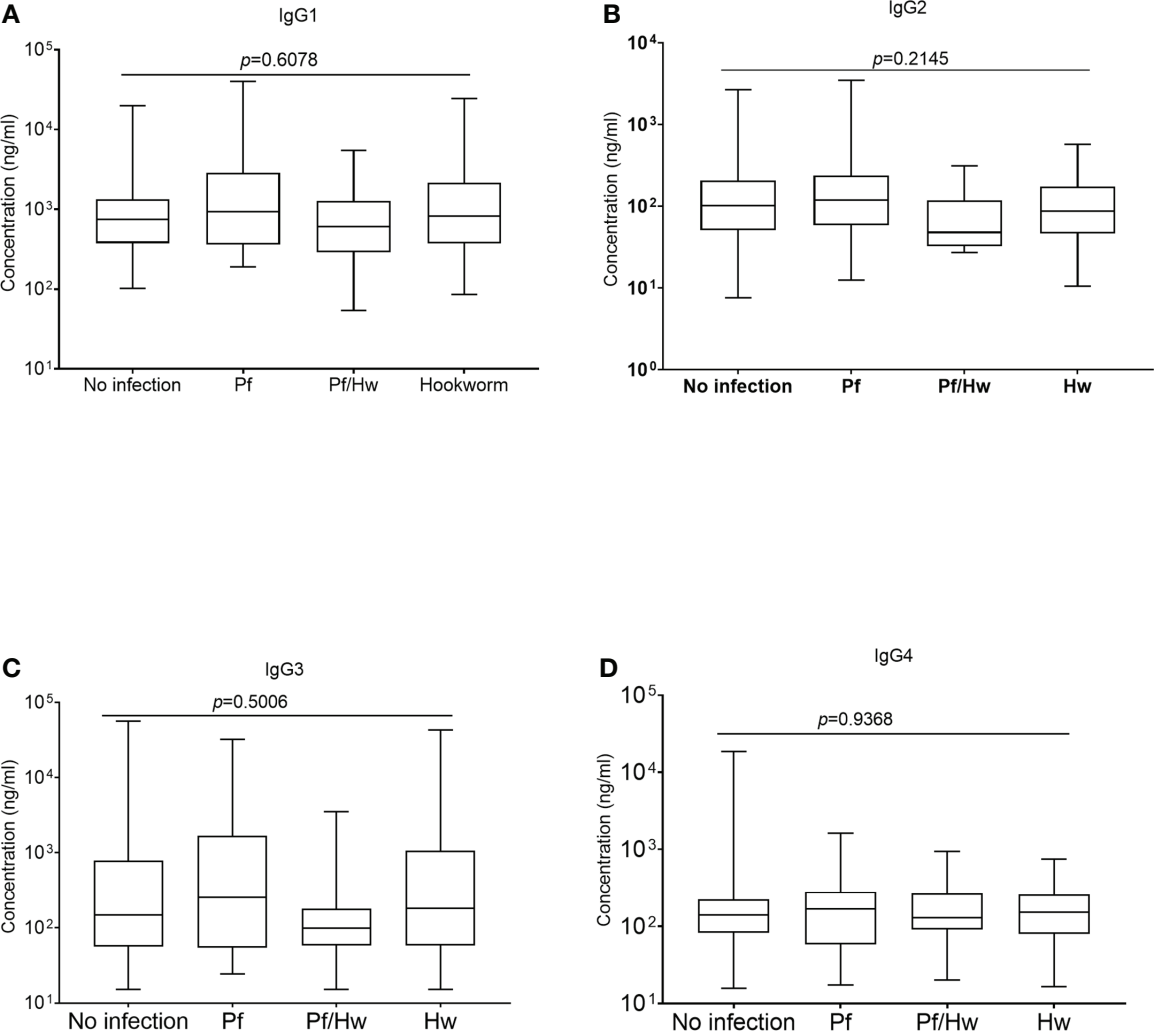
Effect of Hookworm infection on levels of GMZ2-specific IgG subclasses Each box plot represents the median value with the interquartile range, while the whiskers represent the maximum values of antibody concentration in a particular group. Levels of IgG1, IgG2, IgG3 and IgG4 in the various groups are presented in Plots **(A-D)**, respectively.

**Figure 4:**
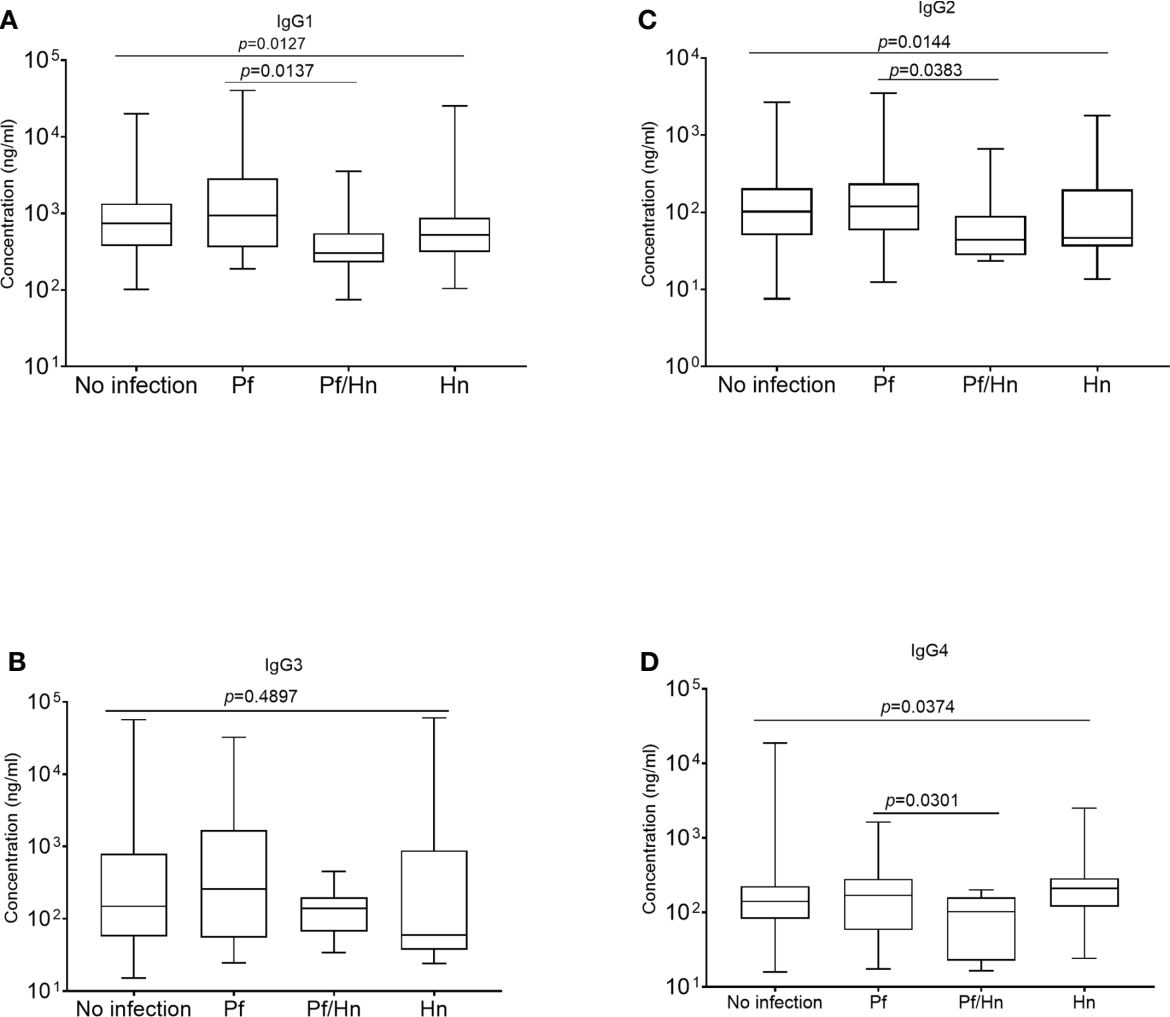
*Hymenolepis nana infection associated with reduced plasma levels of GMZ2-specific IgG subclasses.* The median values are plotted as box plots with the edges corresponding to the interquartile range. The lower whisker of each box indicates the minimum value, while the upper whisker corresponds to the highest antibody value. Levels of IgG1, IgG2, IgG3 and IgG4 in the various groups areare displayed in plots **(A–D)**, respectively.

**Figure 5:**
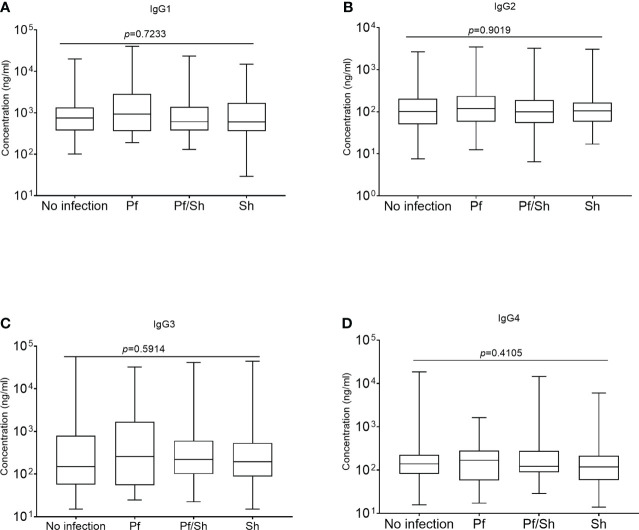
Schistosoma haematobium infection and plasma levels of GMZ2-specific IgG subclasses. Plasma levels of anti-GMZ2 IgG1-4 presented as a box plot with the whiskers of each box indicating the minimum and maximum antibody levels as generated from Kruskal-Wallis analysis on rank variance, which reveals no statistical differences among the groups. Levels of IgG1, IgG2, IgG3 and IgG4 in the various groups are presented in plots **(A–D)**, respectively.

All comparisons were made using the Kruskal-Wallis analysis of variance test, followed by correction for multiple comparisons and antibody levels presented as the median and interquartile range (IQR). Median levels of the different subclasses of IgG in the various groups of study participants exposed to Hookworm were statistically similar (p>0.05) when compared according to the individual IgG subclass groupings (**Figures 3A–D**). The median level of GMZ2 IgG1 was statistically lower in study participants co-infected with Pf/Hn (301.0 ng/ml, IQR, 228.1-550.0) than in Pf (935.6 ng/ml, IQR, 359.4-2866; p=0.0137) infection alone (**Figure 4A**). Similarly, the difference between the median level of GMZ2 IgG2 of the co-infected Pf/Hn (44.24 ng/ml, IQR, 27.72-90.25) and the group without infection (102.0 ng/ml, IQR, 50.67-205.4 *p*=0.0383) is significant (**Figure 4B**). Just like the effect of Pf/Hn co-infections on IgG2 levels, the reduction in the median level of IgG4 in Pf/*H. nana* co-infected individuals (101.7 ng/ml, IQR, 22.48 - 159.2) statistically differed from the Pf alone (168.1 ng/ml, IQR, 57.94 - 281.0; *p*=0. 0301) groups (**Figure 4D**). Conversely, IgG1, IgG2, IgG3 or IgG4 concentrations did not differ statistically (p>0.05) in the various groups exposed to *S. haematobium* infections (**Figures 5A–D**).

### Relationship between levels of antibodies and parasite loads.

Spearman rank correlation analysis, which details the direction and strength of the relationship, if there is any, between two variables with skewed data, revealed a significant positive relationship between the burden of *H. nana* and IgG1, IgG2 and total IgG levels. Similarly, there was a positive relationship between the load of *S. haematobium* and the total levels of IgG (**Table 2**).

**Table 2:** Association between anti-GMZ2 antibodies and parasite load in children.

					Antibodies					
Parasites	IgG		IgG1		IgG2		IgG3		IgG4	
	ρ	p-value	ρ	*p*-value	ρ	*p*-value	ρ	*p*-value	ρ	*p*-value
Pf	0.089	0.362	0.006	0.954	0.078	0.423	0.039	0.687	-0.087	0.375
Hw	0.007	0.947	-0.051	0.652	0.024	0.833	0.039	0.687	-0.089	0.375
Hn	0.299	0.003	0.296	0.003	0.298	0.003	0.17	0.096	0.004	0.967
Sh	0.31	<0.0001	0.052	0.39	0.107	0.073	0.105	0.08	-0.015	0.805

The table was generated from Spearman rank correlation (rho, ρ) analyses which evaluate the relationship between two ranked variables. An absolute ρ value of at least ρ = 0.25 at a p < 0.05 was considered significant. Immunoglobulin (Ig), P. falciparum (Pf), hookworm (Hw), Hymenolepis nana (Hn) and S. haematobium (Sh).

## Discussion

People living in malaria-endemic regions raise antibodies against several malarial antigens, which could be influenced by other infective protozoan and non-protozoan parasites, including helminths. This study evaluated the possible impact of Hookworm, H. nana, and S. haematobium on anti-GMZ2 IgG responses during natural exposure in children aged 5 to 15 years. No relationship was detected between *P. falciparum* density and helminth egg or larvae load which is suggestive of a general lack of effect of *P. falciparum* multiplication on the helminth under consideration. This agrees with an earlier report (25) but contrasts sharply with others (26, 27). As reported, our inability to detect any relationship between *P. falciparum* densities and those of helminth as previously reported may depend on the clinical conditions of the study participants, the degree of infections and the age structure of the study participants as suggested earlier (28). Indeed, the age structure of the study participants in the current report is similar to that of Tuasha et al., which may therefore account for the consistency between these two reports relative the rest which are in conflict with them.

Additional observations from the study reveal that most study participants had anti-GMZ2 IgG and its subclasses to detectable levels in their plasma, just like in other settings (29). Therefore, individuals in Nigeria also raise antibodies against the GLURP and MSP3 subunits measured by antibodies to GMZ2 during natural exposure to malaria to contribute to the development of semi-immunity to malaria. However, it should be noted that, unlike previous reports (22, 24, 30) no statistical difference was detected between the levels of anti-malarial antibodies in *P. falciparum*-positive children and those with *Pf/Sh* co-infection. This observation may be attributed to the impairment of malarial antigen-specific antibody production by *S. haematobium* during coinfection of *P. falciparum* with *S. haematobium.* It is also possible that *S. haematobium* shares cross-reactive epitopes with GMZ2 so that antibodies to *S. haematobium* would react with GMZ2 and vice versa, as previously suggested for the malaria parasite in general (22, 30–32). This may invariably mask any suppressive effect *S. haematobium* might have on antimalarial antibody production, failing to detect any difference, as in this case. This assertion is reinforced by the observed positive relationship between the egg load of *S. haematobium* and antibody levels, especially total IgG levels. Increasing *S. haematobium* egg load could have led to increased secretion of the *S. haematobium* antigens into blood circulation that share cross-reactive epitopes with malaria antigens leading to the positive relationship between the anti-GMZ2 IgG, and *S. haematobium* egg density observed here. This assertion is further reinforced by the apparent lack of any detectable relationship between the density of *P. falciparum* and GMZ2-specific antibodies or between the levels of parasites of *P. falciparum* and *S. haematobium.* However, the effect of this shared epitope phenomenon does not necessarily rule out suppressive attributes of *S. haematobium* infection on the development of antimalarial immunity.

Although *P. falciparum* does not appear to have any effect on *H. nana* intensity and vice versa, co-infection of *P. falciparum* with *H. nana* presented a grievous impairment to the anti-GMZ2 IgG response, which is consistent with experimental evidence that implicates *H. nana*, a human tapeworm, and rodent one (Hymenolepis diminuta) in dampening the immune response (33). This, however, contrasts sharply with a couple of earlier reports (21, 34). The differential outcome may be attributed to the study designs as helminth positive groups reported, and age of the study participants which influences parasites carriage and immunity to either group of parasites (34) could have impacted on the results. More so, *S. haematobium, S. mansoni* and several other helminths have been demonstrated to share cross-reactive epitopes with human malaria antigens (30, 31) and those of rodent malaria (35, 36). The extent to which each helminth contributes to a shared epitope or collectively influence the polyclonal antibody pool is unknown. So lumping all helminths together without filtering may give a different result compared to when filtered. Furthermore, in the current study *H. nana* whose various developmental phases exert discrete effects on immune response, including Th1, Th2 and Th17 cytokines secretion (37, 38) which are important in B lymphocyte biology and antibody production (39, 40) has a strong negative association with IgG levels but did not form part of the helminthic parasites worked on by Santano and colleagues. This may explain the difference in the conclusions arrived at, especially when contrasting effect of helminths on the inflammatory response to malaria have been posted, indicating that different helminths may have divergent effects on the immune response. Our observation, notwithstanding the mechanism by which it is arrived at it is possible the interaction between *H. nana* and the immune system might have affected B lymphocyte biology and/or crosstalk between T and B lymphocytes to give rise to the decrease in the level of these antibodies. This, especially the reduction in the level of cytophilic IgG1, raises questions about the ability of the respective individuals to mount adequate antibody-mediated antimalarial immune responses considering that anti-GMZ2 IgG1 may cooperate with leukocytes (13, 14) to contain an infection. This deficiency may, therefore, account for the disproportionately high prevalence of Plasmodial infections or incidence of malaria in children co-infected with helminths as observed earlier by us and others (17–20).

Whatever parasitic infection is superimposed on the other, dual infections of helminth and *Plasmodium* spp. are common in the study area (17) and the least fancied helminth, *H. nana*, appears to have a far-reaching antagonistic effect on antimalarial immunity. This may influence any intervention implemented to contain each of these parasite types individually. Thus, chemotherapy regimens that can simultaneously control coexisting parasites and other effective control measures may be of utmost importance in the absence of a potent and effective vaccine.

In general, the differential observations made here when data on antibody level from all helminths’ without filtering the parasites were considered as one and compared to those of malaria positive and malaria free groups, or when grouped as intestinal, non-intestinal helminthic infections and compared, or stratified further and looked at individually in comparison to malaria positive and malaria free groups make a strong case for analysis of various data sets independently rather than lumping them together as happened in some cases. Doing so in this report led to and in the future may lead to loss of valuable information hindering our understanding of the true effect the various helminths may have on malaria-specific antibody responses and possibly antibody response to other infectious agents. Indeed, they may not all have the same or similar effect on the anti-malarial specific antibody responses or production as indicated earlier (21, 41–43) or immune response in general as evidenced in human and experimental malaria-helminthiasis co-morbidity (33, 44). Thus, the current report suggests that having sufficient power and looking at the influence of the intestinal parasites on immune response individually may reveal more information and offer us an in-depth understanding of the effect of each parasite on anti-malarial antibody response.

This work, however, has a couple of limitations in our failure to use polymerase chain reaction, a more sensitive technique than Microscopy, to assess infection, especially very light ones. This might have caused us to miss them and misclassified such samples. Additionally, we have not explored the mechanism by which *H. nana* might have dampened the IgG response to malaria during infection as observed as well as not testing the study participants for human immune deficiency virus, a known agent for secondary immunodeficiency, to ascertain their status and correct for it during the analyses. However, it is unlikely that the prevalence of human immune deficiency virus in individuals infected with *H. nana*, especially those with dual *H. nana*/malaria infections, would be different from the rest of the population to compromise the immune response to malaria to give rise to the observation made.

In conclusion, this study suggests that helminthiasis in malaria parasite-infected children could negatively affect antibody production and hence antibody-mediated immune response to malaria. Specifically, *P. falciparum* co-infection with *S. haematobium* or *H. nana* appeared to manipulate the anti-GMZ2 antibody responses involved in developing protective immunity to *P. falciparum*. The current result, therefore, shows that this silent passenger in the gut with worldwide distribution modulates immune response as previously reported (33, 44, 45) and its impact on humans may have far-reaching consequences than previously thought, especially, during co-infections with other pathogens.

## Data availability statement

The original contributions presented in the study are included in the article/**Supplementary Material**. Further inquiries can be directed to the corresponding author.

## Ethics statement

The studies involving human participants were reviewed and approved by The Kwara State Ministry of Health Ethical Committee. Written informed consent from the participants’ legal guardian/next of kin was not required to participate in this study in accordance with the national legislation and the institutional requirements.

## Author contributions

Conceived and designed the experiments: OO and SA. Participants, enrollment: AA. Collected samples: AA and OO. Contributed reagents/materials/tools for analysis: ME, MT, and FN. Performed the experiments: SA and AA. Analyzed the data: SA. Wrote the first draft: SA. Edited and approved the paper: SA, OO, ME, MT, FN, and AA.

## Acknowledgments

We are grateful to the study participants for granting us access to their samples and Dr. Kwadwo Asamoah Kusi of the Immunology Department of the Noguchi Memorial Institute for Medical Research, University of Ghana for the invaluable assistance that was provided. FN is a member of the Central African Network on Clinical Research (CANTAM) and PANDORA-ID-Net, funded by EDCTP and the European Union.

## Conflict of interest

The authors declare that the research was conducted in the absence of any commercial or financial relationships that could be construed as a potential conflict of interest.

## Publisher’s note

All claims expressed in this article are solely those of the authors and do not necessarily represent those of their affiliated organizations, or those of the publisher, the editors and the reviewers. Any product that may be evaluated in this article, or claim that may be made by its manufacturer, is not guaranteed or endorsed by the publisher.

## Supplementary material

Supplementary Figure 1 Helminth infections have no effect on the levels of immunoglobulin G to GMZ2. The data is presented as a box plot with interquartile range and whiskers. Helminths consist of *H. nana*, Hookworm and *S. haematobium* grouped together, Pf, *Plasmodium falciparum* infection, Pf/Helminth, *P. falciparum*-helminth comorbidity and No infection, no parasitic infection. Kruskal-Wallis analysis of rank variance was used to analyze the data followed by Dunn correction for multiple comparisons with a significance level set at p < 0.05.
