# Supplementary figures and images for: Humoral antimalaria immune response in Nigerian children exposed to helminth and malaria parasites
Source: Front Immunol. 2022 Sep 2;13:979727. doi: 10.3389/fimmu.2022.979727 (PMC9494551; doi:10.3389/fimmu.2022.979727)

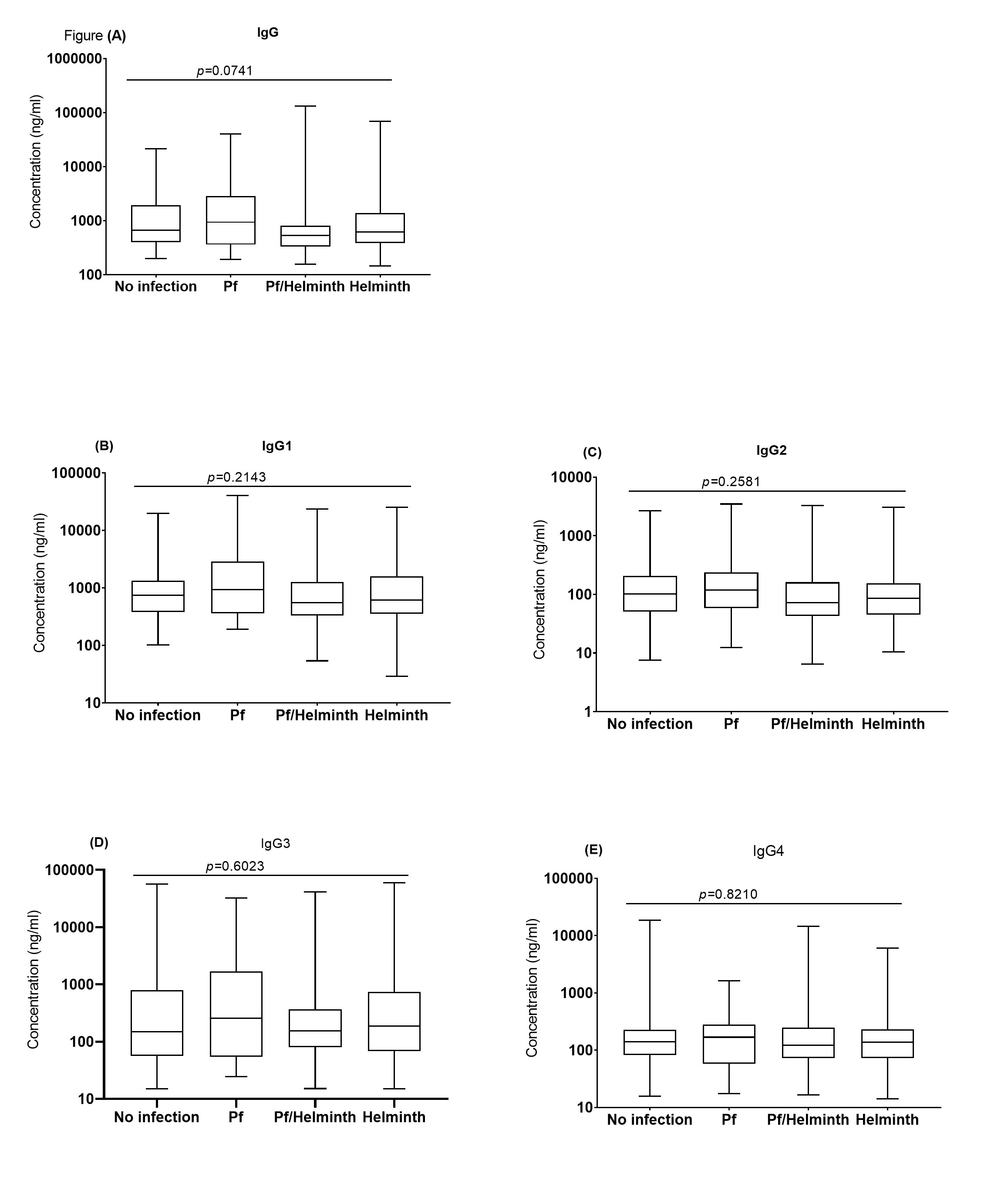

Supplement: Supplementary Figure 1 — Helminth infections have no effect on the levels of immunoglobulin G to GMZ2. The data is presented as a box plot with interquartile range and whiskers. Helminths consist of H. nana, Hookworm and S. haematobium grouped together, Pf, Plasmodium falciparum infection, Pf/Helminth, P. falciparum-helminth comorbidity and No infection, no parasitic infection. Kruskal-Wallis analysis of rank variance was used to analyze the data followed by Dunn correction for multiple comparisons with a significance level set at p < 0.05. [file Image_1.tif]
